# Neck subcutaneous nodule as first metastasis from broad ligament leiomyosarcoma: a case report and review of literature

**DOI:** 10.1186/s12893-020-00951-0

**Published:** 2020-11-25

**Authors:** Fiorella Cazzato, Angela D’Ercole, Graziano De Luca, Francesca B. Aiello, Adelchi Croce

**Affiliations:** 1 Department of Medical, Oral and Biotechnological Sciences, University G. D’Annunzio, Chieti-Pescara, Via dei Vestini, 66100 Chieti, Italy; 2 Surgical Pathology Unit, Santissima Annunziata Hospital, Chieti, Italy; 3 Clinical Pathology Unit, Giuseppe Mazzini Hospital, Teramo, Italy; 4 Department of Medicine and Aging Sciences, University G. D’Annunzio, Chieti-Pescara, Chieti, Italy

**Keywords:** Broad ligament leiomyosarcoma, Head and neck leiomyosarcoma, Atypical uterine smooth muscle tumors, Metastasis, Batson plexus, Case report

## Abstract

**Background:**

Leiomyosarcoma usually develops in the myometrium and is characterized by a high recurrence rate, frequent hematogenous dissemination, and poor prognosis. Metastasis is usually to lungs, liver, and bone, and occasionally to the brain, but seldom to the head and neck region. Primary leiomyosarcoma very rarely arises in the broad ligament.

**Case presentation:**

A 54-year old woman presented to the otolaryngology department with a mass in the right posterior region of the neck 4 years after surgery for a primary leiomyosarcoma of the right broad ligament. The neck mass was removed and found to be a metastatic leiomyosarcoma. Leiomyosarcoma localizations in lungs and liver were absent. Morphological examination showed both the primary and the secondary leiomyosarcomas to have features of low-grade tumors. One year after excision of the neck mass, the patient presented with tachycardia. Echocardiography detected two intracardiac nodules suggestive of metastatic tumors. Chemotherapy was administered; the disease has been stable since then.

**Conclusions:**

We report the first case of broad ligament leiomyosarcoma with the neck subcutaneous region being the first site of secondary involvement. We speculate that the Batson venous plexus might have been the pathway of dissemination.

## Background

Leiomyosarcoma is a malignant tumor derived from smooth muscle [1]. It is most common in women in the fifth decade of life, with the myometrium being the usual site of origin [2]. These tumors accounts for 1% of all gynecological malignancies, 3.7% of uterine malignant tumors, and 25–36% of uterine sarcomas [3]. The incidence of uterine leiomyosarcoma is 0.36 per 100,000 women–years [4]. Leiomyosarcoma of the broad ligament is very rare, with only 24 cases reported to date [5]. Gynecological leiomyosarcoma has poor prognosis because of local invasiveness and a tendency for distant metastasis usually to the liver, lung, and bone [6]. Metastasis to the head and neck region is rare, with only 24 cases reported in the literature [2, 7–12]. The rarity of metastasis to the head and neck increases the likelihood of misdiagnosis or delayed diagnosis [1, 13], particularly when concurrent liver or lung metastases are absent. We report a very rare case of a primary leiomyosarcoma in the broad ligament with first metastasis to the subcutaneous tissue of the neck.

## Case presentation

**Fig. 1:**
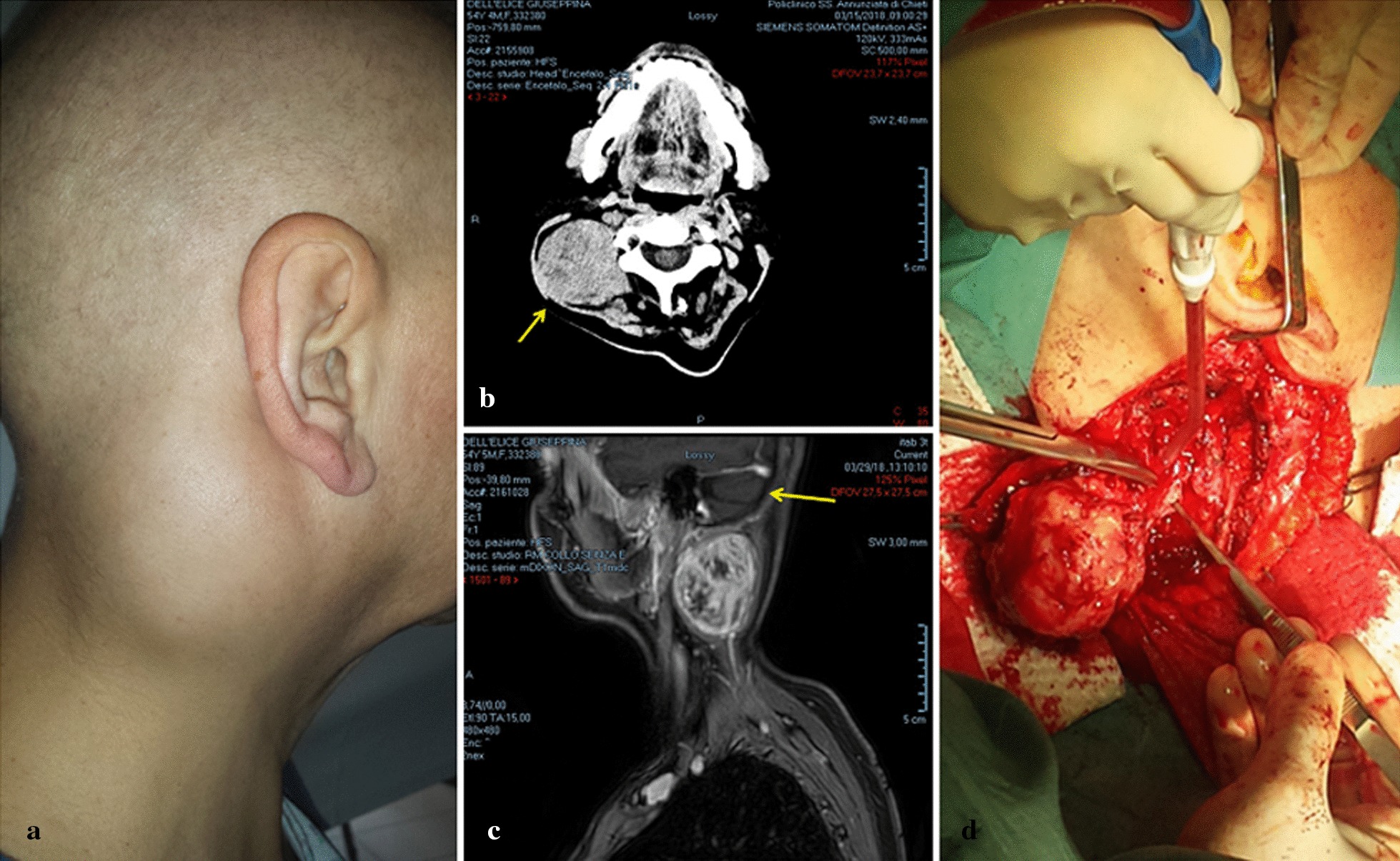
Representative preoperative, CT scan, MRI, and surgical excision images. **a** Preoperative photograph of the mass in the right posterior neck region. **b** CT scan showing a solid mass (indicated by the arrow) in the posterior neck region near C1-C2. **c** MRI image showing a hyper-intense mass displacing paravertebral muscles (arrow), and a cleavage plane between the mass and the sternocleidomastoid muscle. **d** Surgical excision of the mass by posterior neck cervicotomy

A multiparous 54-year-old woman presented to the department of otolaryngology in February 2018 with a round mass, approximately 5 cm in diameter, in the posterior triangle of the right side of the neck (Fig. 1a). The mass was firm, fixed, and painful on palpation. For the past 6 months she had been suffering from neck pain, which was interpreted as torticollis and treated with analgesics without relief. Whole-body contrast-enhanced computed tomography (CT) scan revealed a solid 5.5 × 4.5 cm mass in the right posterior neck region near C1 and C2, without bone infiltration or enlargement of the carotid space lymph nodes (Fig. 1b); there was also a 0.8-cm subcutaneous nodule near the anterolateral arch of the right third rib and the axillary cavity. The lungs and liver were normal. Contrast-enhanced magnetic resonance imaging (MRI) of the neck showed a heterogeneously hyperintense mass displacing the paravertebral muscles, and a distinct cleavage plane between the mass and the sternocleidomastoid muscle (Fig. 1c). Fine needle aspiration cytology (FNAC) was performed twice, however, only few lymphoid cells and erythrocytes were observed.

About 4 years earlier, in October 2013, the patient underwent surgical removal of a large nodule present in the right broad ligament, and homolateral salpingo-oophorectomy (Additional file 1). The nodule was diagnosed as leiomyosarcoma. One month later the patient was treated with total abdominal hysterectomy, left salpingo-oophorectomy and radiotherapy. The histological analyses did not show any other leiomyosarcoma localizations, thus, the diagnosis of primary leiomyosarcoma of the right broad ligament was formulated (TNM: pT1, pN0, pM0). In 2017, she had had leiomyosarcoma relapse on the scar of the previous surgical procedure, indicative of shedding of sarcomatous cells, probably during surgery of the leiomyosarcoma nodule, and received one cycle of adriamycin plus olaratumab, a monoclonal antibody approved by the Food and Drug Administration in 2016 for the treatment of metastatic soft tissue sarcomas.

The rarity of metastasis of leiomyosarcoma to the head and neck region, and the absence of lung and liver metastases, led us to suspect a primary neck tumor.

**Fig. 2:**
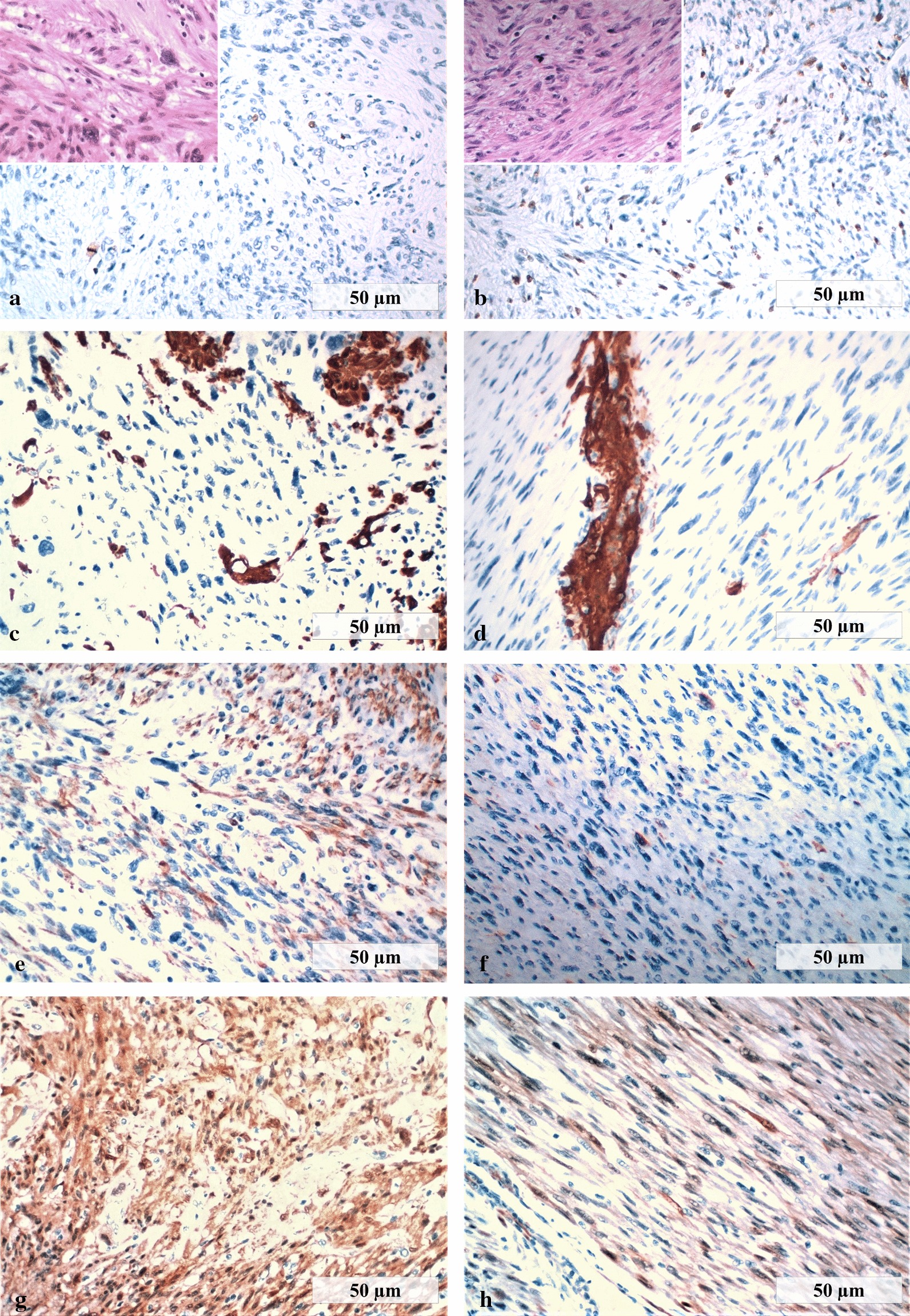
Immunohistochemical evaluation of broad ligament and neck leiomyosarcomas. **a** MIB-1 immunoreactivity in the primary LMS. **b** MIB1 immunoreactivity in the neck LMS. Insets in **a** and **b** show correspondent hematoxylin–eosin stainings. **c**, Smooth muscle actin immunoreactivity in the primary LMS. **d**, Smooth muscle actin immunoreactivity in the neck LMS. **e**, Desmin immunoreactivity in the primary LMS. **f**, Desmin immunoreactivity in the neck LMS. G, p16 immunoreactivity in the primary LMS. **h**, p16 immunoreactivity in the neck LMS. (**a**–**h**: × 200)

The neck mass (Fig. 1d) and the subcutaneous nodule near the right axilla were excised. The morphological features of the neck lesion matched those of the earlier broad ligament leiomyosarcoma, and so secondary leiomyosarcoma was diagnosed. The primary leiomyosarcoma was a large (19 × 16.8 × 6 cm) light-gray mass, with the cut section showing yellow areas of softening and occasional foci of hemorrhage. Microscopically, a few atypical pleomorphic elements were interspersed between bundles of uniform spindle cells and necrotic areas (Fig. 2a, inset). The neck mass was a lobulated gray nodule, 5.7 cm in diameter, presenting whorled and necrotic areas on cut section, without macroscopic and microscopic evidence of skeletal muscle infiltration. Necrosis was present in < 50% of the samples of both the primary and the secondary tumors. Mitotic figures (MF) were infrequent: < 3 MF/10 HPF and < 5 MF/10 HPF in the primary and in the secondary leiomyosarcomas, respectively; no atypical MF were present. On immunohistochemical examination, both lesions showed MIB-1 nuclear immunoreactivity of < 5% (Fig. 2a, b), focal marked smooth muscle actin and desmin immunoreactivity, marked diffuse p16 immunoreactivity (Fig. 2c–h), and weak diffuse vimentin immunoreactivity (not shown). Both tumors were negative for estrogen receptor (ER), p53, CD117, DOG-1, Bcl2, and cyclin D1. According to the French Federation of Cancer Centers Sarcoma Group (FNCLCC) grading system, the score of the mitotic count (score 1 for a mitotic count of 0–9 MF/10 HPF, 2 for 10–19 MF/10 HPF and 3 for > 20/10 HPF) combined with the scores of the differentiation degree and the necrosis extension establish LMS diagnosis and grade. The broad ligament sarcoma was classified as a grade 1 well-differentiated leiomyosarcoma. The metastatic leiomyosarcoma also displayed a well-differentiated morphology.

**Fig. 3:**
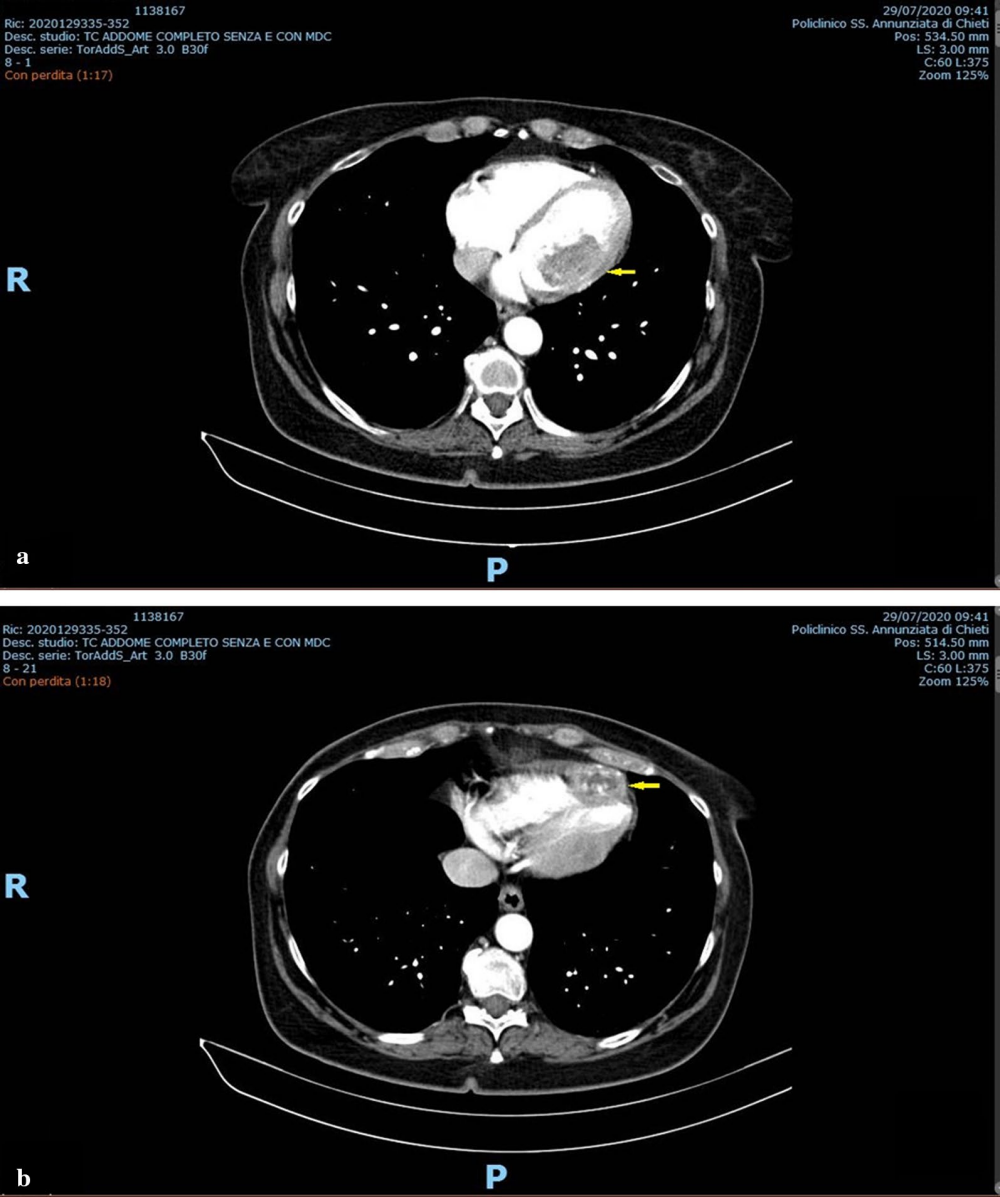
Chest CT scan (July 2020). Intracardiac masses exhibiting inhomogeneous intensity. **a** Nodule on the lateral wall of the left ventricle (arrow). **b** Nodule at the apex of the right ventricle (arrow)

The postoperative course was uneventful. She continued olaratumab therapy and remained in good condition until 13 months after the surgery, when a chest CT scan detected two intracardiac masses. Positron emission tomography (PET)-CT scan and MRI confirmed the presence of the intracardiac masses. Echocardiography showed two hyperechogenic inhomogeneous intracardiac nodules: one (21 × 33 mm) in the lateral wall of the left ventricle and the other (17 × 15 mm) at the apex of the right ventricle. The patient experienced tachycardia**.** Biopsy was not performed because of the high surgical risk. She was treated with three cycles of chemotherapy (dacarbazine and gemcitabine). At follow-up in November 2019, the nodules were found to have increased in size (37 × 28 mm and 44 × 25 mm, respectively) thus monotherapy with pazopanib was introduced. In February 2020, the nodule at the right ventricle apex had grown further, therefore a chemotherapy regimen with paclitaxel alone was initiated. After the fifth course, in April 2020, no further increase in size of either nodule was observed. Heart rhythm had also returned to normal. The disease has been stable since then. The results of a CT scan, performed in July 2020, are shown in Fig. 3.

## Discussion and conclusions

Our patient was unusual in that the subcutaneous tissue of the neck was the first metastatic site of a leiomyosarcoma of the broad ligament. In a previous review of a large cohort of 25,000 sarcomas, only 65 (0.26%) sarcomas were found to have metastasized to the skin or subcutaneous tissue, and in only 17 patients was it the first manifestation of the disease [14]; moreover, only 3 patients, unsorted for sarcoma type, had the neck as the initial site of metastasis.

It is important to distinguish a primary subcutaneous leiomyosarcoma from a secondary leiomyosarcoma because treatment and prognosis are very different [15]. Only five cases of secondary head and neck leiomyosarcoma—without hepatic or pulmonary metastases—have been documented to date [8, 15, 16, 18, 19]. In two of those cases, a retroperitoneal leiomyosarcoma was the primary neoplasm [16, 19] while, in the other three, the primary was a uterine leiomyosarcoma [8, 15, 18]. In two cases [15, 16], the metastases were the first presentation of the disease, while in the other three cases—as in our patient—the primary leiomyosarcoma had been previously diagnosed.

The rarity of spread of a uterine leiomyosarcoma to the neck region, without involvement of lung and liver metastases, led us to speculate on the route of dissemination. One possible route is the Batson vertebral venous plexus, which consists of four valveless networks that surround the vertebral column, connecting the cervical, thoracic, abdominal, and pelvic regions [17, 20]. In 1940, the otolaryngologist Oscar V. Batson classified human veins in the caval, portal, pulmonary, and vertebral systems, He showed that increased intrathoracic/abdominal pressure occurring, for instance, during the Valsalva manoeuvre, diverted the flow in the vertebral plexus [20]. This was later confirmed by the demonstration that positive pressure ventilation during general anaesthesia could drive cells from a tumor into the Batson plexus [21, 22], indicating that the cells could indeed bypass the pulmonary and/or the hepatic filters [23, 24]. Batson plexus drains blood from the pelvis, including blood from the broad ligament, but it is an unlikely route of spread for retroperitoneal tumors because it does not receive tributaries from this region [16]. In our patient, the leiomyosarcoma cells might have spread via Batson plexus to reach the neck region, the axillary cavity, the abdominal wall, and even the heart. Echocardiography and CT scan results, and the positive response to the therapy supported the metastatic origin of the cardiac nodules.

Cardiac secondaries from leiomyosarcoma are extremely rare [25]. Their frequency is considerably lower than in leukemia, lymphoma, melanoma, lung, breast, and gastric cancer [26]. In a large study including 2600 cases of cardiac metastasis only six originated from LMS [26]. LMS cardiac metastases preferably occur in long-term surviving patients [27], as observed in our patient.

Histopathology is useful for leiomyosarcoma grading, and some immunohistochemical markers have established prognostic value. Diffuse cellular pleomorphism, extensive necrosis, numerous MF and MIB-1 positive cells, and p53 positivity indicate high-grade tumor and worse prognosis [28, 29].

Leiomyosarcoma has a high recurrence rate and a tendency for hematogenous dissemination. Median survival is 20.6 months [30]. In a large cohort study of uterine leiomyosarcoma, 50% of cases were metastatic at time of diagnosis [32]. Further, primary leiomyosarcoma of the broad ligament is reported to be especially aggressive [31]. In our patient, although the primary tumor was large, histological samples showed < 50% coagulative necrosis, well-differentiated cytomorphology, low number of MF and MIB-1-positive cells, and p53 negativity. These features may explain why distant metastases became evident 4 years after excision of the primary. Both the primary and secondary leiomyosarcomas in our patient were ER negative and p16 immunoreactive. ER negativity has recently been reported to be associated with relapse [30]. Generally, inactivation of the tumor suppressor protein p16 has a negative prognostic value [35, 36]; however, this protein has other less studied functions, and several tumors overexpress it [35]. Overexpression occurs in approximately 5% of leiomyomas and 50% of leiomyosarcoma [30, 33–35]. These figures, confirmed by a recent meta-analysis [37], suggest a negative correlation with differentiation and prognosis.

To conclude, we report for the first time an extremely unusual case of well-differentiated leiomyosarcoma of the broad ligament with the neck subcutaneous tissue as the first site of metastasis, without lung or liver involvement. This unique case shows that even a low-grade leiomyosarcoma of the broad ligament has a concrete metastatic potential and that alternative routes of spread exist.

## Supplementary information

**Additional file 1:** Timeline care.

## Data Availability

The datasets used/and or analyzed in this study are available from the corresponding authors on reasonable request.

## Abbreviations

CT: Computed tomography
MRI: Magnetic resonance imaging
FNAC: Fine needle aspiration cytology
MF: Mitotic figures
HPF: High power field
ER: Estrogen receptor
PET-CT scan: Positron emission tomography-computed tomography scan
