# Supplementary material for: Neck subcutaneous nodule as first metastasis from broad ligament leiomyosarcoma: a case report and review of literature
Source: BMC Surg. 2020 Nov 25;20:297. doi: 10.1186/s12893-020-00951-0 (PMC7687730; doi:10.1186/s12893-020-00951-0)
Supplement: Supplementary file 1 — Additional file 1: Timeline care. [file 12893_2020_951_MOESM1_ESM.docx]

| October 2013 | Diagnosis of primary LMS of the right broad ligament, after salpingo-oophorectomy and total abdominal hysterectomy. |
| --- | --- |
| February 2018 | Surgical removal of neck subcutaneous mass and diagnosis of LMS metastasis. The patient continued olaratumab as monotherapy. |
| March 2019 | RMI and PET detection of two intracardiac masses. Patient started chemotherapy with dacarbazine and gemcitabine |
| November 2019 | Increase in size of cardiac nodules.  The patient started therapy with pazopanib |
| February 2020 | Cardiac masses increased in size. The patient replaced pazopanib with paclitaxel as monotherapy. |
| April 2020 | The disease is stable after 5 cycles of paclitaxel. |

TIMELINE (CARE)
